# 3D Printing of Octacalcium Phosphate Bone Substitutes

**DOI:** 10.3389/fbioe.2015.00081

**Published:** 2015-06-08

**Authors:** Vladimir S. Komlev, Vladimir K. Popov, Anton V. Mironov, Alexander Yu. Fedotov, Anastasia Yu. Teterina, Igor V. Smirnov, Ilya Y. Bozo, Vera A. Rybko, Roman V. Deev

**Affiliations:** ^1^A.A. Baikov Institute of Metallurgy and Materials Science, Russian Academy of Sciences, Moscow, Russia; ^2^Institute of Laser and Information Technologies, Russian Academy of Sciences, Moscow, Russia; ^3^Human Stem Cells Institute, Moscow, Russia; ^4^A.I. Evdokimov Moscow State University of Medicine and Dentistry, Moscow, Russia; ^5^A.I. Burnazyan Federal Medical Biophysical Center of FMBA of Russia, Moscow, Russia; ^6^Institute of Carcinogenesis, N.N. Blokhin Russian Cancer Research Center, Moscow, Russia; ^7^Kazan Federal University, Kazan, Russia

**Keywords:** 3D printing, tricalcium phosphate, octacalcium phosphate, ceramics, bone graft, *in vivo* test, osteoconductivity

## Abstract

Biocompatible calcium phosphate ceramic grafts are able of supporting new bone formation in appropriate environment. The major limitation of these materials usage for medical implants is the absence of accessible methods for their patient-specific fabrication. 3D printing methodology is an excellent approach to overcome the limitation supporting effective and fast fabrication of individual complex bone substitutes. Here, we proposed a relatively simple route for 3D printing of octacalcium phosphates (OCP) in complexly shaped structures by the combination of inkjet printing with post-treatment methodology. The printed OCP blocks were further implanted in the developed cranial bone defect followed by histological evaluation. The obtained result confirmed the potential of the developed OCP bone substitutes, which allowed 2.5-time reducing of defect’s diameter at 6.5 months in a region where native bone repair is extremely inefficient.

## Introduction

Biocompatible synthetic grafts and/or tissue engineering constructions prevail over conventional approaches based on autologous, allogenous, or xenogenous bone tissue. However, complex structure and properties of natural bone limit the spectrum of synthetic materials and fabrication techniques that could be used as custom-designed implants or scaffolds for bone defects replacement or guided bone regeneration. Currently, this problem might be solved via a 3D printing technique (Bergmann et al., 2010; Bose et al., 2013; Popov et al., 2014).

Synthetic calcium phosphates’ (CP) chemical similarity to the natural bone mineral content allows to apply it successfully as bone substitutes among a variety of other materials (ceramics, bioglasses, polymers, and their combinations). A number of CP biomaterials with different phase compositions [hydroxyapatite (HA), tricalcium phosphate (TCP), ion-substituted CP, etc.] and several formulations have been developed over the last few decades (Bohner, 2010; Dorozhkin, 2011). Most of these biomaterials are used in clinical practice as granules, cements, or porous blocks (Bohner, 2010). TCP ceramics are a reliable, osteoconductive, and biodegradable material, and it is already commercially available (Suba et al., 2006; Horowitz et al., 2009; Stavropoulos et al., 2010). However, it has recently been demonstrated that octacalcium phosphate (OCP) spherical microporous ceramic granules are three times more effective than TCP for bone marrow mesenchymal stromal cells differentiation *in vivo* (Zorin et al., 2014). Additionally, OCP ceramics possess osteogenic features of interest, e.g., stimulate lamellar bone formation for 2 months after *in vivo* implantation. About 4–5 months after OCP ceramics implantation in clinical trials computer tomography (CT) and histological examinations of patient’ biopsies of the bone defect revealed new bone formation (Komlev et al., 2014). Thus, OCP-based implants are the most promising for 3D printing.

There are two major techniques currently available for ceramics 3D printing. The first one is mixing of CP powders or agglomerates with polymers, e.g., collagen, followed by their chemical solidification, and finally high-temperature processing (Detsch et al., 2011; Rath et al., 2012). Another technique comprises sacrificial inverse matrix printing, its infiltration with ceramic slurry, and burning out the negative (Guo et al., 2009; Schumacher et al., 2010).

The main concept of our work is based on an approach, which involved chemical interaction between initial CP powder, such as TCP, and binder liquid (“ink”), such as diluted phosphoric acid, followed by chemical treatment of the printed dicalcium phosphate dihydrate (DCPD) structures in solutions. Therefore, a support material burning out was not necessary that prevented undesirable contamination and risk of OCP decomposition.

## Materials and Methods

### Chemicals and reagents

All reagents were purchased from Sigma-Aldrich: high-purity-grade calcium nitrate (Cat. No: 13477-34-4), ammonium carbonates (Cat. No: 506-87-6), ammonium phosphate monobasic (Cat. No: 7722-76-1), potassium carbonate-sodium carbonate mixture (Cat. No: 10424-09-6), and sodium acetate (Cat. No: 127-09-3).

Tricalcium phosphate powder was synthesized in an aqueous medium by slow addition of diammonium phosphate [(NH_4_)_2_HPO_4_] solution into calcium nitrate [Ca(NO_3_)_2_4H_2_O] solution, containing NH_4_OH, under constant stirring. The pH of the mixture was about 7 with Ca/P molar ration of 1.5/1. After total addition of the reactants, the suspension was filtered, dried at 80°C and sintered at 700°C for 2 h. TCP agglomerates with mean size 40–80 μm were used for printing. TCP crystal aggregates were prepared by light grinding using a pestle and then passing through a standard testing sieve.

1.0% aqueous solution of salts of phosphoric acid (pH 4.75) was used as “ink” for the 3D printer. The buffer solution was prepared by dissolving in water of 1.5 ± 0.1M sodium acetate, 1.0 ± 0.1M phosphoric acid, and 0.15 ± 0.01M glutamic acid.

### Printing process

**Figure 1:**
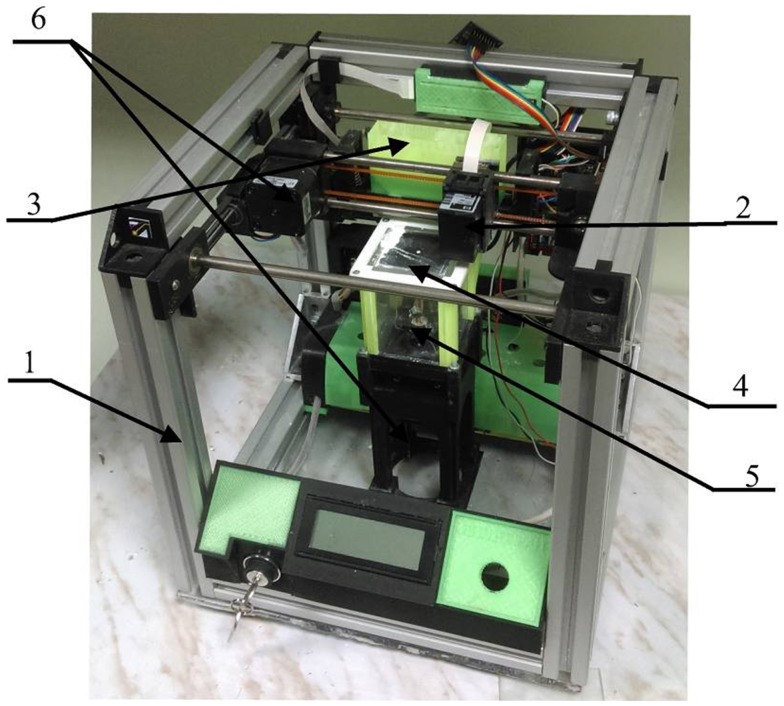
**Custom-designed 3D printer**. 1, 3D printer frame; 2, printing head; 3, stuffer with spreader; 4, Z-piston; 5, building box; 6, stepper motors.

To develop the process of layer-by-layer 3D printing of OCP bone substitutes, we used our custom-designed 3D printer (shown in Figure 1). Briefly, it consists of a ceramic powder stuffer with a spreader (3) and *Z*-piston (4) inside a building box (5), providing TCP powder layer up and down movement according to computer-controlled algorithm. Bidirectional (*X*–*Y*) positioning system with a printing head (2) made from modified cartridges for standard inkjet printer (HP C6602A, Hewlett-Packard, USA). Both *Z*-piston and *X*–*Y* movement of printing head (“ink” container with nozzles producing droplet size ca. 40 pL) are driven with stepper motors (6) with *X*–*Y*–*Z* accuracy of ca. 40 μm. Flexibility of our 3D printer design comprising relatively small building envelope (60 mm × 60 mm × 60 mm) permits testing of small amounts of powder materials. It makes possible fast and inexpensive study of both new process techniques and new material combinations.

Initially, a 3D dataset of desired ceramic model (a bulging disk with a diameter 20 mm and 16 perforations of 1 mm in diameter) in the STL format is uploaded to the system. The recoating mechanism (3) carries an amount of fine (40–80 μm) TCP powder to the building box, creating a thin (ca./200 μm) layer of powder on the top of the *Z*-piston plane (4) inside the building box (5) at room temperature. The liquid binder (200 pL/point) is ejected from the printing head onto the powder layer with linear speed up to 10 mm/s, wetting individual cross-section. When the layer is completed, *Z*-piston moves down by the thickness of a layer (ca./200 μm) and a new layer of powder is deposited on the printed one. These process steps are repeated until the whole sample is formed within the powder bed. The surrounding powder material supports the ceramic model during the printing process. Thus, there is no necessity for further maintenance structures for such features as overhangs and undercuts. Upon completion, the sample is removed from the building box, cleaned using an air blower, and air dried at room temperature for further chemical treatment.

### Post-treatment of printed structures

After printing, the samples were placed in aqueous solution, which was prepared by dissolving of 115 g of NH_4_H_2_PO_4_ in 500 mL of distilled water at room temperature. The pH of the solution was 4.1 ± 0.1. The samples were kept for 168 h at 40°C. After incubation, the samples were thoroughly washed in distilled water at least 10 times, dried at 37°C and placed in a second solution, which was prepared by dissolving 95.2 g of CH_3_COONa in 700 mL of distilled water at 40°C and pH 8.2 ± 0.2. The samples were again kept for 168 h at 40°C, thoroughly washed in distilled water at least five times and dried overnight at 37°C (Komlev et al., 2014).

### Material characterization

Phase composition was analyzed by conventional X-ray diffraction (XRD) technique [Shimadzu XRD-6000 (Japan), Ni-filtered CuKα_1_ target, λ = 1.54183 Å]. The samples were scanned from 2θ = 3°–60° with a 0.02° step a preset time of 5 s.

Scanning electron microscopy (SEM) apparatus (Tescan Vega II, Czech Republic), working in secondary and backscattered electron modes, was used for microstructure studies. The samples were sputter-coated with a 25 nm-thick gold layer prior imaging to impart electrical conductivity to the surfaces.

The compressive strength of samples was evaluated in accordance with the ISO standard 9917E. The 3D printed cylindrical samples were about 12 mm in height and 6 mm in diameter. Five samples for each point were used. Compression testing was carried out using an Instron 4082 (Bucks, UK) testing machine operating at a crosshead speed of 1 mm × min^−1^. Statistical analysis was performed using SPSS software, version 17.0 (Statistical Package for Social Sciences, SPSS Inc., USA). The means and SD of compressive strength were calculated.

### *In vivo* test: Cranial model

All manipulations with rabbits (*n* = 5) were carried out in accordance with Animal Welfare Act in the vivarium of the A.I. Burnazyan Federal Medical Biophysical Center. The experimental study protocol was approved by the Moscow Interuniversity Ethics Committee (protocol No. 12–13 from December 12, 2013).

### Implantation of the 3D printed bone graft

The animals underwent intramuscular premedication (Sol. Atropini sulfatis 0.1% – 0.04 mg/kg; Sol. Cefazolini 1.0 – 25 mg/kg) and sedation (Sol. Zoletili 100 – 15 mg/kg) before positioning on the operating table special for rabbits and fixation: lying on an abdomen with the paws extended and attached. A linear 3 cm skin incision was performed to a periosteum over sagittal suture from occipital protuberance to the frontal area under local anesthesia (Sol. Ultracaini 1.7 mL). Local anesthetic was applied into the skin and underlying tissues infiltrating periosteum. Soft tissues were lateralized, and the cranial bones surface was exposed. Previously prepared sterile template (disk 20 mm in diameter) was positioned in the center of calvaria from occipital to frontal bone and was used for marking of the defect edges. After that, a template was removed, and osteotomy was made with a bur to form the full-thickness defect (diameter 20 mm) keeping a dura mater uninjured and preserving the small fragments of inner cortical bone (1 mm × 1 mm) in the 1, 5, 7, and 11 o’clock positions as the points of support. Bleeding from the damaged sagittal sinus was stopped by coagulation. 3D printed block exactly corresponding to the defect form was implanted on the retained cortical bone fragments. Surgical wound was closed on multiple tissue levels with interrupted sutures (MonoSyn 4/0). No pain management was required postoperatively. The rabbits were deceased at 6.5 months after surgery. Each calvaria with the bone defect region was removed, fixed in 4% neutral formalin, and subjected to further studies.

### Computer tomography

Explanted material underwent a CT after 3 days of fixation. Scanning parameters were as follows: voxel size 8 μm, 80 kV, and 2 mA.

The tomograms were analyzed with Planmeca Romexis viewer (Planmeca Oy, Finland). Additionally, we carried out a manual segmentation of the area with implanted 3D printed samples and calculated a density (HU) of selected zone in a standard module of 3D-Slicer (NHI, USA).

### Histological examination

Histological slices were made according to the standard procedure after decalcification of the explanted calvaria in the “Biodek-R” solution (Bio-Optica, Italy). All sections were made strictly in the frontal plane through the center of the implant with preservation of parietal bones fragments attached to each side of the 3D printed block (length of the sample was about 25 mm). Histological sections were stained with hematoxylin and eosin and subjected to scanning (Mirax scanner, Carl Zeiss, Germany). Digital images of the histotopograms were analyzed at various magnifications.

## Results and Discussion

3D printing technique based on cement powders is an effective and inexpensive method for individual and complex bone substitute’s fabrication since there is neither support materials burning out nor organic solvent required (Castilho et al., 2014). Setting and hardening processes during 3D printing are based on two types of interaction, which depend on the raw materials used. The first is an acid–base reaction with the formation of a neutral compound. The second one is the hydrolysis reaction of the metastable CP. Both resulted in an adhesive effect between the particles. The final phases of the cement product are apatite or DCPD (Khalyfa et al., 2007; Gbureck et al., 2008; Klammert et al., 2010). An approach was reported utilizing further cement matrix formation consisting of a mixture of HA/TCP by following heat treatment (Castilho et al., 2014). CP materials printed with these techniques are biocompatible and possess certain osteoconductive properties.

**Figure 2:**
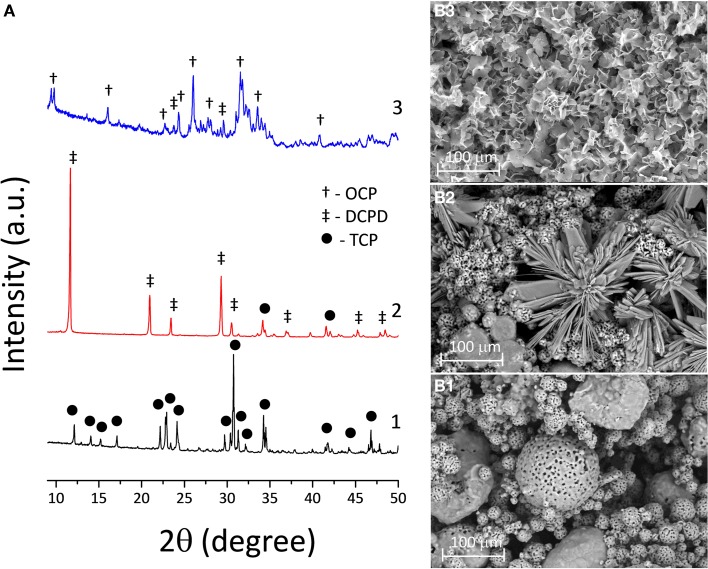
**(A)** XRD chart of the transformation of 3D printed TCP (1) to DCPD samples soaked in calcium nitrate solution during 168 h (2) and to OCP samples in sodium acetate during 168 h (3). SEM photomicrographs of 3D printed samples: **(B1)** TCP (pre-treated material), **(B2)** DCPD (after soaking in calcium nitrate solution at 168 h), and **(B3)** OCP (after soaking in sodium acetate at 168 h).

**Figure 3:**
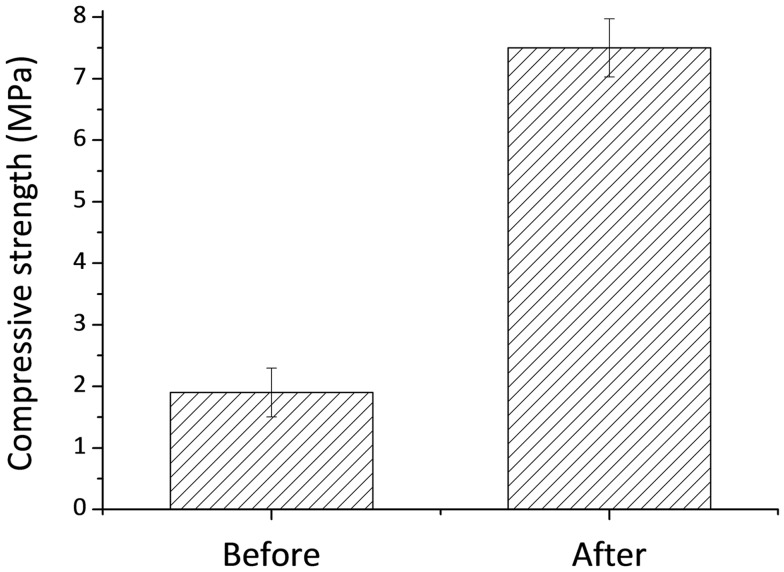
**Compressive strength of 3D printed samples before and after chemical treatment**.

Our work is a combination and further development of the processes involving chemical interaction between initial CP powder, such as TCP and binder liquid (“ink”) such as diluted phosphoric acid (Popov et al., 2014), followed by chemical treatment of the printed DCPD structure with chemical solutions at physiological temperatures. It is known that TCP upon treatment with phosphoric can be used as bone cement forming DCPD, which further can be transformed into OCP (Heughebaert et al., 1983). The 3D printing process of TCP powders with phosphoric acid is based on a hydraulic setting reaction leading to DCPD crystallization, and thus to layer-crossing bonding of the powder finally resulting in the formation of a 3D structure. The printed samples consist of unreacted TCP and certain amounts of DCPD (Figure 2A). SEM photomicrographs of both TCP and DCPD phases are presented in Figures 2B1,B2. Size of the unreacted TCP particles was 5–15 μm (Figure 2B1). The DCPD crystals had a flower-like morphology. The width of the DCPD crystals was in range from 1 to 50 μm, and their thickness ranged from a fraction of few microns (Figure 2B2). Compressive strength of the 3D printed samples is shown in Figure 3 and was only about 2.5 MPa.

To this end, the chemical and phase composition of the 3D printed samples is to be fixed by completing acid–base reaction with DCPD compound formation. Further hydrolysis reaction of the metastable CP results in the formation of OCP phase and an adhesive effect between particles. This process is represented in Figure 2A, where the transformation from TCP to DCPD was completed after 168 h of soaking in the solution. The obtained 3D printed samples with DCPD phase composition were transformed into OCP of soaking in sodium acetate, according to XRD (Figure 2A). XRD of the OCP samples devoted predominant OCP phase with (100) reflection at 2θ = 4.9°. However, the modified samples contained small amounts of unreacted DCPD, as well as new nucleated HA phase (after 168 h of soaking in sodium acetate). High intensity of diffraction peaks indicate high crystallinity of OCP materials (Figure 2A). OCP plates were needle-like 2–5 μm long and 1–2 μm wide (Figure 2B3).

From the data that were presented in Figure 2, the following mechanism can be established: in the initial stage, the pH was low due to the presence of phosphoric acid, and the reaction between Ca_3_(PO_4_)_2_ and H_3_O^+^ yielded Ca^2+^ ions. The Ca^2+^ ions reacted with ${\mathrm{HPO}}_{4}^{2-}$ ions, which formed CaHPO_4_⋅2H_2_O. The further increase of the values of pH of the solution during the post-treatment leads to OCP nucleation and growth.

Compressive strength of 3D printed samples and post treated structures is shown in Figure 3. The compressive strength of the post treated material increased with time from 2.5 MPa up to about 7.5 MPa at *P* ≤ 0.005. The increase in the compressive strength after treatment can be explained by the formation of new OCP crystals within 3D printed samples, which improve the bonding between particles. These OCP samples were used for *in vivo* experiments.

**Figure 4:**
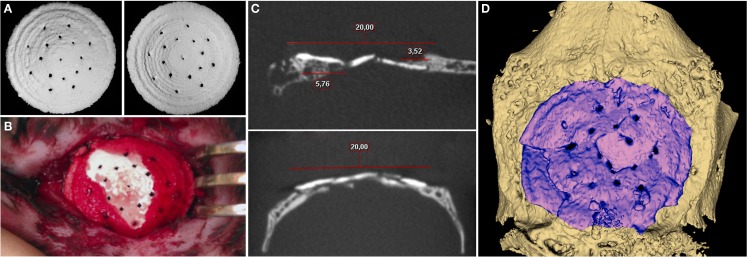
**(A)** General view of 3D printed samples to be implanted. **(B)** Photomicrograph of the cranial defect produced. **(C)** 2D slices in different areas. **(D)** 3D CT reconstruction for 3D printed block at 6.5 months after implantation.

An adequate *in vivo* model for objective evaluation of qualitative and quantitative parameters of substitute’s biological action is utterly important for successful development of 3D printed bone grafts. There are numerous orthotropic animal models: various defects of long bones, mandible, parietal, and frontal bones. However, the majority of bone defects in these models is not large enough or require complex fixation. By choosing an appropriate model for our study, we considered the following criteria: bone defect should be the “maximum-sized” but should not require additional methods of fixation, e.g., osteosynthesis with miniplates, screws, etc., affecting bone regeneration process and, in general, the study results. For this purpose, we developed the original experimental model of a cranial bone defect characterized by large diameter (20 mm) and preservation of four fragments of inner cortical bone in special positions (1, 5, 7, and 11 o’clock) as points for implant support. This model allowed 3D printed block to be optimally immobilized into the bone defects without additional fixation methods (Figures 4A,B).

Postoperatively, there were no signs of acute inflammation, edema, and other adverse events; wound healed by primary intention. According to CT, the peripheral sides of implanted material were fully integrated with surrounding bone tissue (Figure 4). Moreover, there were sites of newly formed tissue, apparently consisting of bone (767.56 ± 145.81 HU), grown centripetally from the temporal edges and tightly adjacent to the inner and external surfaces of the 3D printed block as though enfolding its peripheral parts. A bone regenerate proceeded from occipital bone had the most length that was defined, apparently, by its greater thickness. The average density of the material was 1851.29 ± 58.26 HU 6.5 months after implantation that impeded evaluation of tissue ingrowths into the 3D printed sample. Indirectly, the presence of gaps and sites of superposed edges of the material fragments in the block could identify this process. However, the fragmentation could be related to a mechanical impact of a rabbit’s activity.

**Figure 5:**
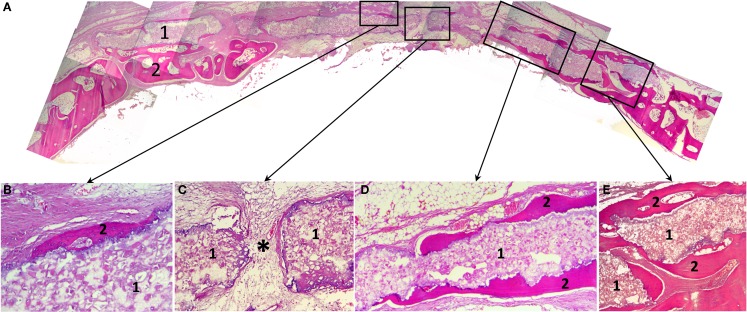
Histological slides of the rabbit’s calvaria bones, slice made in the coronal plane: (A) Histotopogram, including two regions of newly formed bone tissue growing toward each other; (B) the central area, where a fragment of the woven bone tissue formed on the implant surface without fibrous tissue interlayer; (C) the central area with pore in the 3D printed implant a fibrous tissue with single vascular vessels grow out through (*); (D) a fragment of the marginal part of the regenerate where newly formed bone tissue grew directly on the 3D printed block surface; (E) the marginal part of the regenerate having pronounced newly formed bone trabecule retaining the implant. 1, 3D printed block made of octacalcium phosphate; 2, newly formed bone tissue; *vascular vessel. Staining: hematoxylin and eosin, paraffin sections. Magnification: **(A)** ×4, **(C–E)** ×100, **(B)** ×200.

Histological study revealed that 3D printed block was biocompatible: in spite of the large dimensions and the absence of a firm fixation to surrounding structures, material directly contacted with newly formed bone without fibrous encapsulation or even slight connective tissue areas making them apart (Figure 5). In the central part of the defect, no signs of osteogenesis were observed; material here was surrounded by fibrous tissue grown into the block’s pores.

A cross-section shows that a “material–bone tissue” interaction could occur by two ways. First, the bone tissue grows over external and under internal surfaces of the OCP block, but does not penetrate it as the structure was not porous that preserved mechanical qualities of the material. Second, regenerating calvarial bones can grow under the block using it as a conductor. The second way is probably associated with surgical implantation technique.

General feature of this 3D printed block is blood vessels, cells of granulation tissue, as well as osteogenic cells permeability. Bone formation occurred in the sites of close contact with the material that could also be found in the central part of the defect, without any apparent connection with regenerate grown from the edges. In crack sites, the material fragments injured regenerating tissues that led to local giant cell reaction. This finding together with abovementioned confirmed that the gaps were caused by animal activity rather than tissues ingrowths.

The printed implants supported bone regeneration that allowed 2.5-time reducing of defect’s diameter at 6.5 months in a region where native bone repair is extremely inefficient.

## Conclusion

The results of our study demonstrate that combination of 3D inkjet printing with post-treatment methodology is a promising approach to overcome current limitations in effective and fast fabrication of individual constructions for guided bone regeneration. We proposed and developed a relatively simple route and materials for 3D printing process, targeted to production of complexly shaped and structured OCP bone substitutes. We showed experimentally that 3D inkjet powder printing is a suitable technique for custom-designed critical size OCP bone grafts production.

## Conflict of Interest Statement

The authors declare that the research was conducted in the absence of any commercial or financial relationships that could be construed as a potential conflict of interest.

## References

[B1] Bergmann C. Lindner M. Zhang W. Koczur K. Kirsten A. Telle R. (2010). 3D printing of bone substitute implants using calcium phosphate and bioactive glasses. J. Eur. Ceram. Soc. 12, 2563–2567. 10.1016/j.jeurceramsoc.2010.04.037

[B2] Bohner M. (2010). Resorbable biomaterials as bone graft substitutes. Mater. Today 13, 24–30. 10.1016/S1369-7021(10)70014-6

[B3] Bose S. Vahabzadeh S. Bandyopadhyay A. (2013). Bone tissue engineering using 3D printing. Mater. Today 12, 496–504. 10.1016/j.mattod.2013.11.017

[B4] Castilho M. Moseke C. Ewald A. Gbureck U. Groll J. Pires I. (2014). Direct 3D powder printing of biphasic calcium phosphate scaffolds for substitution of complex bone defects. Biofabrication 6, 015006. 10.1088/1758-5082/6/1/015006 24429776

[B5] Detsch R. Schaefer S. Deisinger U. Ziegler G. Seitz H. Leukers B. (2011). In vitro-osteoclastic activity studies on surfaces of 3D printed calcium phosphate scaffolds. J. Biomater. Appl. 26, 359–380. 10.1177/0885328210373285 20659962

[B6] Dorozhkin S. (2011). Calcium orthophosphates: occurrence, properties, biomineralization, pathological calcification and biomimetic applications. Biomatter 1, 121–164. 10.4161/biom.18790 23507744 PMC3549886

[B7] Gbureck U. Hölzel T. Biermann I. Barralet J. E. Grover L. M. (2008). Preparation of tricalcium phosphate/calcium pyrophosphate structures via rapid prototyping. J. Mater. Sci. Mater. Med. 19, 1559–1563. 10.1007/s10856-008-3373-x 18236137

[B8] Guo D. Xu K. Han Y. (2009). The in situ synthesis of biphasic calcium phosphate scaffolds with controllable compositions, structures, and adjustable properties. J. Biomed. Mater. Res. 88, 43–52. 10.1002/jbm.a.31844 18257062

[B9] Heughebaert J. C. Zawacki S. J. Nancollas G. H. (1983). The growth of octacalcium phosphate on beta tricalcium phosphate. J. Cryst. Growth 63, 83–90. 10.1016/0022-0248(83)90431-1

[B10] Horowitz R. A. Mazor Z. Miller R. J. Krauser J. Prasad H. S. Rohrer M. D. (2009). Clinical evaluation alveolar ridge preservation with a beta-tricalcium phosphate socket graft. Compend. Contin. Educ. Dent. 30, 588–590. 19998726

[B11] Khalyfa A. Vogt S. Weisser J. Grimm G. Rechtenbach A. Meyer W. (2007). Development of a new calcium phosphate powder-binder system for the 3D printing of patient specific implants. J. Mater. Sci. Mater. Med. 18, 909–916. 10.1007/s10856-006-0073-2 17216579

[B12] Klammert U. Gbureck U. Vorndran E. Rödiger J. Meyer-Marcotty P. H. Kübler A. C. (2010). 3D powder printed calcium phosphate implants for reconstruction of cranial and maxillofacial defects. J. Craniomaxillofac. Sur. 8, 565–570. 10.1016/j.jcms.2010.01.009 20206538

[B13] Komlev V. S. Barinov S. M. Bozo I. I. Deev R. V. Eremin I. I. Fedotov A. Y. (2014). Bioceramics composed of octacalcium phosphate demonstrate enhanced biological behavior. ACS Appl. Mater. Interfaces 6, 16610–16620. 10.1021/am502583p 25184694

[B14] Popov V. K. Komlev V. S. Chichkov B. N. (2014). Calcium phosphate blossom for bone tissue engineering. Mater. Today 2, 96–97. 10.1016/j.mattod.2014.01.015

[B15] Rath S. N. Strobel L. A. Arkudas A. Beier J. P. Maier A. K. Greil P. (2012). Osteoinduction and survival of osteoblasts and bone-marrow stromal cells in 3D biphasic calcium phosphate scaffolds under static and dynamic culture conditions. J. Cell. Mol. Med. 16, 2350–2361. 10.1111/j.1582-4934.2012.01545.x 22304383 PMC3823428

[B16] Schumacher M. Deisinger U. Detsch R. Ziegler G. (2010). Indirect rapid prototyping of biphasic calcium phosphate scaffolds as bone substitudes: influence of phase composition, macroporosity and pore geometry on mechanical properties. J. Mater. Sci. Mater. Med. 21, 3119–3127. 10.1007/s10856-010-4166-6 20953674

[B17] Stavropoulos A. Windisch P. Szendröi-Kiss D. Peter R. Gera I. Sculean A. (2010). Clinical and histologic evaluation of granular beta-tricalcium phosphate for the treatment of human intrabony periodontal defects: a report on five cases. J. Periodontol. 81, 325–334. 10.1902/jop.2009.090386 20151813

[B18] Suba Z. Takács D. Matusovits D. Barabás J. Fazekas A. Szabó G. (2006). Maxillary sinus floor grafting with β-tricalcium phosphate in humans: density and microarchitecture of the newly formed bone. Clin. Oral Implants Res. 17, 102–108. 10.1111/j.1600-0501.2005.01166.x 16441791

[B19] Zorin V. L. Komlev V. S. Zorina A. I. Khromova N. V. Solovieva E. V. Fedotov A. Y. (2014). Octacalcium phosphate ceramics combined with gingiva-derived stromal cells for engineered functional bone grafts. Biomed. Mater. 9, 055005. 10.1088/1748-6041/9/5/055005 25167539

